# Association between Hyperhomocysteinemia and Thyroid Hormones in Euthyroid Diabetic Subjects

**DOI:** 10.1155/2015/196379

**Published:** 2015-06-21

**Authors:** Yun Zhang, Qiong Wang, Quanzhong Li, Ping Lu

**Affiliations:** Department of Endocrinology, Henan Province People's Hospital and Zhengzhou University People's Hospital, 7 Weiwu Road, Zhengzhou, Henan 470003, China

## Abstract

*Objectives*. The concept now emerging is that higher thyroid-stimulating hormone (TSH) and lower thyroid hormone levels within the euthyroid range may adversely affect atherosclerosis. The present study aimed to investigate the potential associations between thyroid parameters and hyperhomocysteinaemia in a cohort of euthyroid diabetic subjects.* Material and Methods*. Two hundred and seventy-three euthyroid diabetic subjects (167 males and 106 females) were consecutively recruited in this cross-sectional study. Clinical and biomedical data was collected.* Results*. TSH level was higher in females than males. Compared to normal-homocysteine group, hyperhomocysteinaemia group was more likely to be elderly, males, with longer diabetes history, and with lower diastolic blood pressure. Free thyroxine (FT4) level was lower in hyperhomocysteinaemia group than in normal-homocysteine group; however, it was not statistically significant. Adjusted for age, sex, body mass index, duration of diabetes, blood pressure, fasting glucose, total cholesterol, and triglyceride in logistic regression analyses, hyperhomocysteinaemia was significantly correlated with FT4 (*P* = 0.021). No significant association was found with TSH or free triiodothyronine. When analyzed in subjects with TSH < 2.5 uIU/mL separately, we got similar results.* Conclusions*. In conclusion, we identified a relation between hyperhomocysteinemia and FT4 in a group of euthyroid diabetic patients.

## 1. Introduction

Thyroid dysfunction is a risk factor for cardiovascular disease, yet most subjects at risk for cardiovascular disease are euthyroid in the clinical setting. The concept now emerging is that higher thyroid-stimulating hormone (TSH) and lower thyroid hormone levels within the euthyroid range might adversely affect (subclinical) atherosclerosis [1–3]. Studies have revealed that free thyroxine (FT4), free triiodothyronine (FT3), and TSH are significantly associated with lipids profile in the euthyroid population regardless of gender [4–6]. However, studies in hypothyroid individuals show that elevated cholesterol and lipoprotein levels could not fully explain the increased cardiovascular morbidity. Other pathogenic factors might be involved.

Homocysteine is a sulfur-containing amino acid naturally found in human blood, which could induce endothelial injury, oxidative stress, smooth muscle hypertrophy and oxidation of low density lipoprotein cholesterol, and the process of atherosclerosis and cardiovascular diseases [7]. It has often been shown to be related to occlusive vascular disease independent of other known risk factors [8]. An earlier study indicated that an increase in plasma homocysteine level of 4 *μ*mol/L conferred a 40% increase in relative risk for coronary heart disease compared with healthy controls [9]. Hyperhomocysteinemia in overt hypothyroidism had been found in many studies [10, 11]. Even, in subclinical hypothyroidism patients, homocysteine concentration was reported to be higher as compared with euthyroid controls [12]. Furthermore, homocysteine level had been reported to be associated with carotid intima media thickness in patients with clinical hypothyroidism [13].

Diabetes mellitus, in particular type 2 diabetes, is also known to dramatically increase risk of cardiovascular diseases [14]. Thyroid disease and diabetes are the two most common endocrinopathies in the clinical practice, and they often coexist. Thyroid dysfunctions are more frequent in diabetic patients than in the general population [15]. Thyroid dysfunction had been associated with unfavorable changes in several metabolic parameters like lipid profile, which might amplify the cardiovascular disease risk in type 2 diabetes. Moreover, the absolute homocysteine level was also found to be higher in diabetic patients [16, 17].

Taking into account the considerations mentioned above, we hypothesized that effects of low thyroid function on increase of homocysteine level might extend into the euthyroid range, especially in diabetic individuals. The aim of this study was to investigate the potential associations between TSH and thyroid hormone levels within the normal range and hyperhomocysteinaemia in a cohort of euthyroid type 2 diabetic subjects.

## 2. Materials and Methods

### 2.1. Patients

Two hundred and seventy-three euthyroid type 2 diabetes subjects (167 males and 106 females) were consecutively recruited at inpatient clinic of Department of Endocrinology, Henan Province People's Hospital. Type 2 diabetes was diagnosed according to ADA 2009 criteria [18]. Euthyroidism was defined as TSH, FT3, and FT4 levels within their normal reference ranges (see the next paragraph). Exclusion criteria included type 1 diabetes, latent immune diabetes of the adults, gestational diabetes, and other types of diabetes [18], pregnancy, neoplasms, and any major medical condition in the 6 months preceding the study (i.e., liver, kidney, and heart failure). In particular, subjects with a previous history of thyroid diseases, such as overt hyper/hypothyroidism and thyroid cancer, were excluded. Subjects taking medications affecting thyroid hormone levels (such as thyroid supplementation and antithyroid agents, IFNc, amiodarone, lithium, corticosteroids, etc.) were also excluded. Informed consent was obtained from all participants. The present study was approved with the Institutional Ethnics Committee in Zhengzhou University.

### 2.2. Clinical, Anthropometric, and Laboratory Measurements

Clinical data was collected from all participants. Weight and height were measured with the subjects wearing light clothing and no shoes; the body mass index (BMI) was also calculated (kg/m^2^). Blood pressure was measured twice in the sitting position on the right arm using a sphygmomanometer (Erka), after at least 5 min of rest. Whole blood samples were drawn in the morning after an overnight fasting for the measurement of serum TSH, FT3, and FT4 levels, as well as biochemical indicators. Serum TSH, FT3, and FT4 levels were measured using chemiluminescence tests (Siemens Advia Centaur XP). The normal ranges were as follows: FT3 3.5–6.5 pmol/L; FT4 11.5–22.7 pmol/L; and TSH 0.55–4.78 uIU/mL. Homocysteine was assayed using enzymatic methods (Roche Diagnostics), with reference values for adult male and females between 5 and 15 *μ*mol/L, together with fasting blood glucose (FBG), total cholesterol (TC), and triglyceride (TG).

### 2.3. Statistical Analyses

The data analysis was performed using SPSS version 18.0. All data was expressed as means ± standard deviation (SD). All the subjects were divided into two groups according to homocysteine level: hyperhomocysteinaemia group (>15 *μ*mol/L) and normal-homocysteine group (≤ 15 *μ*mol/L). Two-sided *t*-tests and Pearson's Chi-square tests served to analyze the differences in means and proportions between the two groups. Logistic regression analysis was performed to evaluate the association of hyperhomocysteinaemia with thyroid parameters, adjusted for age, sex, BMI, duration of diabetes, systolic blood pressure (SBP), diastolic blood pressure (DBP), FBG, TC, and TG. Furthermore, subjects with TSH <2.5 uIU/mL were analyzed separately. And statistical significance was defined as *P* < 0.05.

## 3. Results and Discussion

Table 1 showed the clinical characteristics of the 273 subjects. The mean age was 54.50 ± 12.03 years (range 23 to 82), and the mean BMI was 25.23 ± 3.69 kg/m^2^ (range 17.5 to 43.0). Homocysteine, DBP, TG, and FT3 levels were lower in females than in males, and TSH levels were higher. No difference was found in age, BMI, FBG, FT4, or duration of diabetes (Table 1).

Compared to the normal-homocysteine group, the hyperhomocysteinaemia group was more likely to be elderly, males and have longer diabetes history and lower DBP levels. FT4 level was lower in hyperhomocysteinaemia group than in the normal-homocysteine group; however, it was not statistically significant. Adjusted for age, sex, BMI, duration of diabetes, blood pressure, FBG, TC, and TG in logistic regression analyses, hyperhomocysteinaemia was significantly correlated with FT4 (*P* = 0.021), age (*P* = 0.002), sex (*P* = 0.000), DBP (*P* = 0.015), and TG (*P* = 0.035). No significant association was found with FT3 (*P* = 0.736) or TSH (*P* = 0.061) (Table 2).

In 2003, the National Academy of Clinical Biochemistry (NACB) recommended lowering the upper reference limit of TSH to 2.5 uIU/mL based on a large-scale epidemiological survey that revealed that more than 95% of normal individuals had TSH levels < 2.5 uIU/mL and that those with higher TSH levels were likely to have various thyroid disorders [19]. So we did an analysis in subjects with TSH < 2.5 uIU/mL separately and got similar results: hyperhomocysteinaemia was still correlated with FT4 (*P* = 0.049) in logistic regression analysis (Table 3).

So, in this study, we demonstrated an association between hyperhomocysteinaemia, an independent risk factor of cardiovascular diseases, and lower FT4 levels, indicative of low normal thyroid function, in a group of euthyroid diabetic patients. No association was found with FT3 or TSH. Even when TSH was restricted to smaller than 2.5 uIU/mL, we got similar results. The relationship between thyroid hormones and atherosclerosis in the euthyroid population has garnered much interest recently. These observations were in line with previous studies in overt hypothyroid patients. Study by Diekman et al. indicated that FT4 was an independent determinant of homocysteine concentrations; log (FT4) levels and age accounted for 28% of the variability of homocysteine [20]. Bamashmoos et al. reported that homocysteine was significantly positively correlated with TSH and negatively correlated with FT4 and there were no significant correlations with FT3 [21]. However, another study reported that homocysteine significantly negatively correlated only with FT3 [22].

Thyroid hormones were recognized as catabolic hormones and they regulated various processes of metabolism. Homocysteine metabolism might be influenced by thyroid hormone through two different pathways. Firstly, thyroid hormone could influence the activity of hepatic enzymes involved in the remethylation pathway of homocysteine, methionine synthase, and methylenetetrahydrofolate reductase [23–25]. Secondly, low level of thyroid hormone probably reduces glomerular filtration rate leading to increased creatinine and homocysteine levels [20, 26, 27]. Our study suggested that, even in euthyroid subjects with normal thyroid hormones and TSH, thyroid function might influence the homocysteine metabolism, leading to hyperhomocysteinaemia. And we could suppose that homocysteine might be involved in the association between thyroid function and atherosclerosis, even in euthyroid subjects.

Several methodological aspects and limitations of our study need to be considered. First of all, the causal relationship could not be inferred from this study because it was cross-sectional in nature. Secondly, only FBG level was used to represent glucose control, which was not good enough; however, glycated hemoglobin was not measured. Furthermore, in type 2 diabetes patients, homocysteine levels might be influenced by other variables, such as serum creatinine levels, comorbidities, and/or hypoglycemic therapies. However, all these were not considered in this study. Also, as only 273 subjects were recruited, the statistical power of the present study was limited. Further investigations are needed to understand the association of homocysteine levels with thyroid parameters and the intimate mechanisms.

## 4. Conclusions

In conclusion, we identified an association between hyperhomocysteinemia and FT4 in a group of euthyroid type 2 diabetes patients for the first time. A longitudinal study is needed to assess the effects of the variation in thyroid hormone levels within the euthyroid range in the development of hyperhomocysteinaemia.

## Figures and Tables

**Table 1:** Clinical characteristics of the subjects.

	Total	Male	Female	*P*
*n*	292	167	106	
Age (years)	54.50 ± 12.03	53.57 ± 12.48	55.96 ± 11.20	0.110
Duration of diabetes (years)	8.35 ± 7.31	8.82 ± 7.60	7.62 ± 6.81	0.185
BMI (kg/m^2^)	25.23 ± 3.69	25.44 ± 3.15	24.90 ± 4.40	0.239
SBP (mmHg)	134.48 ± 16.09	135.01 ± 16.15	133.64 ± 16.03	0.494
DBP (mmHg)	82.92 ± 9.85	84.46 ± 10.27	80.49 ± 8.66	0.001
TC (mmol/L)	4.79 ± 1.07	4.76 ± 1.09	4.84 ± 1.03	0.524
TG (mmol/L)	1.99 ± 1.77	2.18 ± 2.06	1.70 ± 1.14	0.029
FBG (mmol/L)	8.78 ± 3.81	8.88 ± 3.91	8.63 ± 3.66	0.607
Homocysteine (*μ*mol/L)	14.73 ± 8.67	16.23 ± 10.00	12.36 ± 5.21	0.000
FT3 (pmol/L)	4.48 ± 0.52	4.61 ± 0.55	4.27 ± 0.40	0.000
FT4 (pmol/L)	16.33 ± 2.36	16.44 ± 2.31	16.16 ± 2.45	0.346
TSH (uIU/mL)	2.15 ± 0.96	2.05 ± 0.93	2.30 ± 1.00	0.037

**Table 2:** Correlation of thyroid parameters with hyperhomocysteinaemia.

	Hyperhomocysteinaemia	Normal-homocysteine	*P*	*P*′
*n*	100	173		
Homocysteine (*μ*mol/L)	21.59 ± 11.02	10.76 ± 2.38	0.000	
Age (years)	57.87 ± 12.81	52.55 ± 11.14	0.001	0.002
Sex (males/females)	78/22	89/84	0.000	0.000
Duration of diabetes (years)	9.60 ± 7.86	7.63 ± 6.89	0.039	0.678
BMI (kg/m^2^)	24.92 ± 3.20	25.41 ± 3.94	0.286	0.221
SBP (mmHg)	134.25 ± 17.29	134.61 ± 15.40	0.858	0.777
DBP (mmHg)	81.08 ± 10.91	83.98 ± 9.05	0.019	0.015
TC (mmol/L)	4.80 ± 1.15	4.79 ± 1.02	0.931	0.907
TG (mmol/L)	2.19 ± 2.40	1.88 ± 1.27	0.224	0.035
FBG (mmol/L)	8.39 ± 3.28	9.00 ± 4.08	0.196	0.115
FT3 (pmol/L)	4.49 ± 0.59	4.47 ± 0.49	0.785	0.736
FT4 (pmol/L)	16.15 ± 2.42	16.64 ± 2.24	0.098	0.021
TSH (uIU/mL)	2.23 ± 1.02	2.10 ± 0.93	0.260	0.061

*P*: differences between the two groups by Student's *t*-tests.

*P*′: association with hyperhomocysteinaemia by logistic regression analyses and with homocysteine level (1 = hyperhomocysteinaemia, 0 = normal-homocysteine) as dependent variables and independent variables as follows: age, sex, BMI, duration of diabetes, BMI, SBP, DBP, TC, TG, FBG, FT3, FT4, and TSH.

**Table 3:** Correlation with hyperhomocysteinaemia in patients with TSH < 2.5 uIU/mL.

	Hyperhomocysteinaemia	Normal-homocysteine	*P*	*P*′
*n*	69	124		
Homocysteine (*μ*mol/L)	21.59 ± 11.62	10.78 ± 2.41	0.000	
Age (years)	57.93 ± 13.09	51.35 ± 10.43	0.000	0.003
Sex (males/females)	57/12	68/56	0.000	0.000
Duration of diabetes (years)	10.01 ± 8.62	7.08 ± 6.61	0.016	0.586
BMI (kg/m^2^)	25.00 ± 3.22	25.56 ± 3.98	0.318	0.393
SBP (mmHg)	134.35 ± 18.54	134.44 ± 15.56	0.972	0.747
DBP (mmHg)	80.72 ± 11.05	83.66 ± 9.37	0.052	0.131
TC (mmol/L)	4.78 ± 1.07	4.71 ± 0.97	0.634	0.334
TG (mmol/L)	2.09 ± 2.02	2.04 ± 1.36	0.842	0.518
FBG (mmol/L)	8.21 ± 3.00	9.29 ± 4.29	0.041	0.063
FT3 (pmol/L)	4.55 ± 0.62	4.49 ± 0.47	0.508	0.822
FT4 (pmol/L)	16.28 ± 2.42	16.91 ± 2.12	0.060	0.049
TSH (uIU/mL)	1.64 ± 0.47	1.61 ± 0.50	0.678	0.850

*P*: differences between the two groups by Student's *t*-tests.

*P*′: association with hyperhomocysteinaemia by logistic regression analyses and with homocysteine level (1 = hyperhomocysteinaemia, 0 = normal-homocysteine) as dependent variables and independent variables as follows: age, sex, BMI, duration of diabetes, BMI, SBP, DBP, TC, TG, FBG, FT3, FT4, and TSH.
